# Identification of lncRNA biomarkers in hepatocellular carcinoma by comprehensive analysis of the lncRNA-mediated ceRNA network

**DOI:** 10.3389/fgene.2022.832952

**Published:** 2022-08-29

**Authors:** Dingde Ye, Yaping Liu, Yanuo Chen, Guoqiang Li, Beicheng Sun, Jin Peng, Qingxiang Xu

**Affiliations:** ^1^ Medicine School of Southeast University Nanjing Drum Tower Hospital, Nanjing, China; ^2^ The First Affiliated Hospital of Anhui Medical University, Hefei, China; ^3^ School of Life Science and Technology, Southeast University, Nanjing, China; ^4^ Department of General Surgery, Affiliated Drum Tower Hospital, Medical School, Nanjing University, Nanjing, China

**Keywords:** hepatocellular carcinoma, competing endogenous RNA, biomarker, predictive model, lncRNA

## Abstract

Growing evidence implicates that miRNAs can interact with long non-coding RNAs (lncRNAs) to regulate target mRNAs through competitive interactions. However, this mechanism that regulate tumorigenesis and cancer progression remains largely unexplored. Long non-coding RNAs (lncRNAs) act as competing endogenous RNAs (ceRNAs), which play a significant role in regulating gene expression. The purpose of our study was to determine potential lncRNA biomarkers to predict the prognosis of HCC by comprehensive analysis of a ceRNA network. The edgeR package was used to obtain the differentially expressed RNA datasets by analyzing 370 HCC tissues and 50 adjacent non-HCC tissues from The Cancer Genome Atlas (TCGA). Through investigating the differentially expressed between HCC tissues and adjacent non-HCC tissues, a total of 947 lncRNAs, 52 miRNAs, and 1,650 mRNAs were obtained. The novel constructed ceRNA network incorporated 99 HCC-specific lncRNAs, four miRNAs, and 55 mRNAs. Survival analysis identified 22 differentially expressed mRNAs, four miRNAs, and nine lncRNAs which were associated with overall survival (OS) time in HCC (*p* < 0.05), and further exploration was performed to assess the correlation of these differentially expressed genes with tumor stage. The Interpretation of the potential functions of these differentially expressed genes in HCC was realized by Gene ontology (GO) and KEGG pathway enrichment analyses. Seven lncRNAs were confirmed based on univariate Cox regression analysis, lasso COX regression analysis and multivariate Cox regression analysis to construct a predictive model in HCC patients which were related to the prognosis of OS. In summary, ceRNAs contributed to explore the mechanism of tumorigenesis and development, and a model with seven lncRNAs might be potential biomarker to predict the prognosis of HCC. These findings supported the need to studies on the mechanisms involved in the regulation of HCC by ceRNAs.

## Introduction

Hepatocellular carcinoma (HCC) is the sixth most universal malignancy worldwide and third leading cause of tumor-related death (Bray et al., 2018). Despite exploration endeavors and advances in diagnostic and therapeutic strategies, the mortality of HCC remains high. In recent years, the extensive molecular characterization of HCC has revealed the underlying biology in these tumor entities, which offers a potential target for the treatment of HCC (Hachem et al., 2017). Next-generation sequencing technologies which leads to great progress in the comprehension of gene expression. It has largely revealed the cellular transcriptome, biogenesis mechanisms, and the molecular functions of RNAs (Honda et al., 2016). Nevertheless, the interaction mechanism of mRNA/lncRNA/miRNA in the initiation and progression of HCC requires exploration in greater depth.

Long non-coding RNAs (lncRNAs) lacking ability for protein coding are polymerase II transcripts longer than 200 nucleotides (Ganesh et al., 2020). In recent years, evidence has been accumulating to demonstrate that lncRNAs support diverse biological functions (Silva-Fisher et al., 2020), including serving as critical regulators of tumorigenesis and metastasis. Thus, they play important roles in the occurrence and development of diseases, and it has aroused the strong interest of scientific researchers.

MicroRNAs (miRNAs, small non-coding RNA molecules was composed of 19–25 nucleotides) introduce an important impact in multiple cellular functions through the induction RNA silencing and participating in post-transcriptional regulation of gene expression (Li et al., 2017). Memczak et al. initially described the biological functioning of miRNAs. Previous studies have also demonstrated that lncRNAs can act as the ‘sponges’ of microRNA to regulate gene expression (Yu et al., 2017). It has been reported that lncRNA also regulates gene expression through serving as a microRNA (miRNA) sponge, which is also called competitive endogenous RNA (ceRNA) theory.

Recently, ceRNA has been used to explore the mechanism of tumorigenesis and development, and to predict patient prognosis in HCC (Ning et al., 2018). By using RNA sequencing data from TCGA database, we first constructed a ceRNA network to identify HCC-specific RNAs in the present study. Ultimately, a novel combined model was established and validated to predict patient prognosis in HCC.

## Materials and methods

### Data acquisition from TCGA

All data acquired from TCGA are available without restrictions to use, according to the guideline statement from the TCGA website. We extracted clinical data from TCGA database and retrospectively analyzed the characteristics of patients in HCC (https://portal.gdc.cancer.gov/). We extracted RNA transcription data of 420 tissues, including 370 tumor tissues and 50 adjacent non-tumor tissues. The present study was in agreement with the publication guidelines of TCGA database (http://cancergenome. nih.gov/publications/publication guidelines). According to the TCGA database usage statement, no ratification was required from the Ethics Committee.

### Exploration of differentially expressed lncRNAs (DElncRNAs), DEmiRNAs, and DEmRNAs in HCC

We used the ENCODE database (https://www.encodeproject.org, gencode.v22. annotation) to identify mRNAs, lncRNAs, and miRNAs. In this study, mRNAs, lncRNAs, and miRNAs that were not included in the ENCODE database were excluded. Based on the edge R package, differentially expressed genes were acquired based on comparing between cases of HCC and adjacent tumor tissues. The criterion of differential expression in mRNA and lncRNA was specified to logFC ≥2 and false discovery rate (FDR) < 0.05. To prevent the elimination of important differentially expressed miRNAs, the criterion of logFC ≥1 and FDR < 0.05 was specified to selecte significant difference in the miRNA data. For the obtained DEmRNAs, DElncRNAs, and DEmiRNAs, we used the gplots and heatmap packages to draw volcano maps and heat maps. Based on these differentially expressed genes, further exploration was performed to assess the correlation of them with tumor stage (https://www.xiantao.love).

### GO functional analysis and KEGG pathway enrichment analysis of DEmRNAs

In order to accurately annotate gene function, gene ontology (GO) analysis and Kyoto Encyclopedia of Genes and Genomes (KEGG) pathway enrichment analysis of mRNA was performed based on the R language version 4.1.0 ClusterProfiler. Next, org.Hs.eg.db and the enrichplot package to analyze enrichment status. Eventually, all results were visualized through the ggplot2 package. In the GO analysis and KEGG pathway analysis, *p* < 0.05 was regarded as statistically significant.

### Interactions between mRNAs, miRNAs, and lncRNAs

In order to clarify the relationship between the DEmRNAs and DEmiRNAs, three databases that included the miRDB database, miRTarBase database, and TargetScan were used to predict the relationship between miRNAs and target mRNAs, and the intersection of the results predicted through the three databases was used to make the final possible regulate relationship. The miRcode database was used to predict the target site relationship between lncRNAs and miRNAs.

### Construction of the competing endogenous RNA network

Datas of the DEmiRNAs, DEmRNAs and DElncRNAs were applied to construct the competing endogenous RNA (ceRNA) network. Cytoscape v3.8.2 was used to make the results visible.

### Construction of a lncRNA-related prognostic model

The survminer R packages (https://cran.r-project.org/web/views/Survival.html) were used for survival analysis of all differentially expressed lncRNAs in the ceRNA network. The difference in survival of differentially expressed miRNAs and lncRNAs were also analyzed, respectively. The relationship between DElncRNAs and the overall survival (OS) in HCC (*p* < 0.05) was determined by univariate cox regression analysis. The result of the univariate cox regression was applied to multivariate cox regression to establish a regression equation. Risk scores were obtained based on the regression equation. HCC patients were divided into high-risk group and low-risk group through the median risk score. The effect of expression difference of the lncRNAs (high risk vs. low risk) on overall survival (one-, three- and 5-year) was tested by receiver operating curve (ROC). The value of ROC represents the area under the curve (AUC), which was used to indicate prognostic biomarkers in the survival of patient in HCC.

### Statistical analysis

In our study, all datas analyses were carried out using R language version 4.1.0. The identification of DElncRNA and DElncRNA is identificated by univariate, lasso and multivariate Cox regression analyses, which can predict the prognosis of HCC. Based on this, we could then propose interventional measures to improve the prognosis of patients. For the OS analysis, the method of Kaplan-Meier was used to calculated the survival rate, and then compared the survival curves using the log-rank test.

## Results

### Identifying DElncRNAs, DEmiRNAs, and DEmRNAs in HCC

**FIGURE 1:**
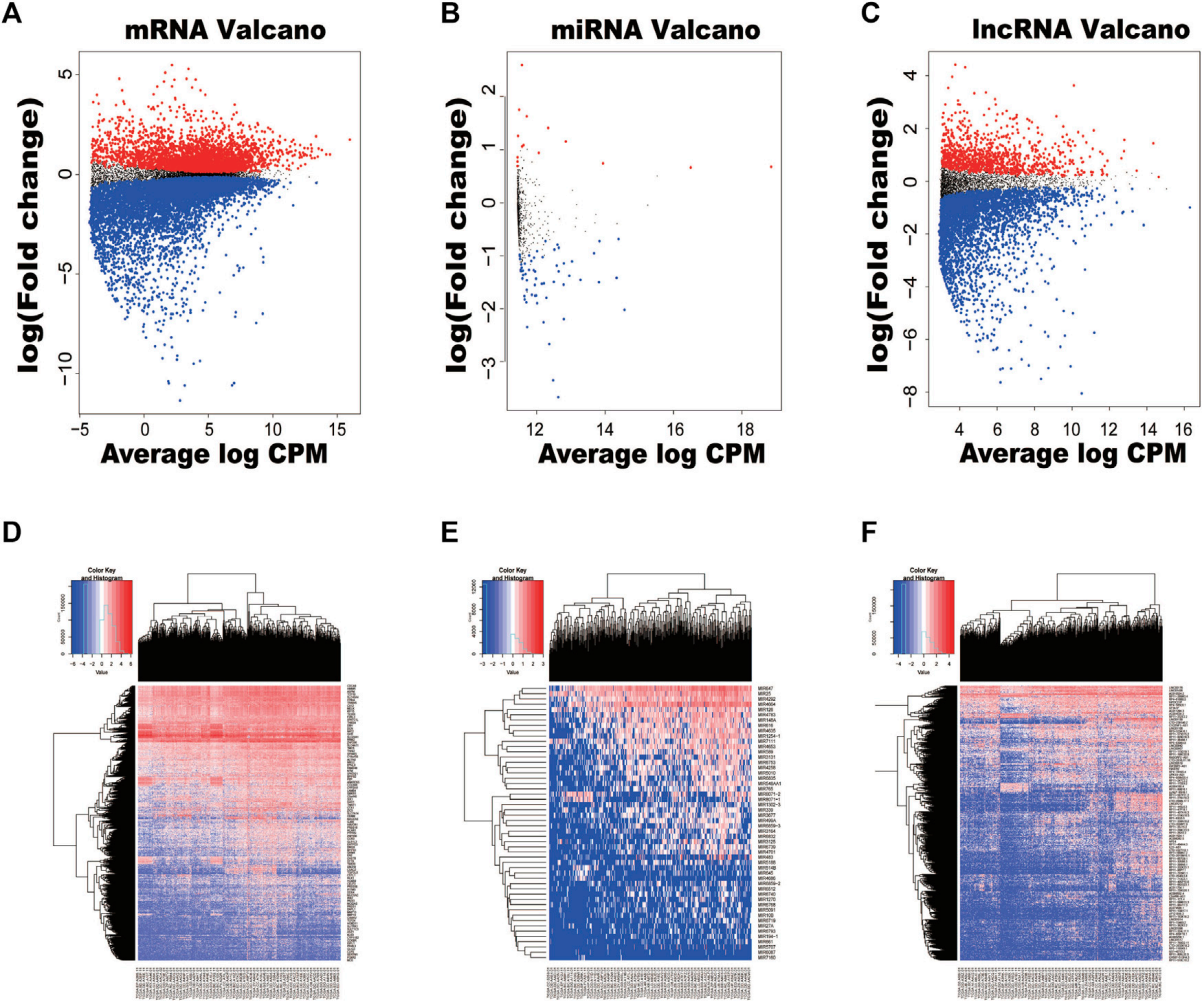
Volcano plots and heatmaps of differentially expressed RNAs between hepatocellular carcinoma (HCC) tissues and normal tissues. **(A)** Volcano plot of HCC-specific mRNAs. **(B)** Volcano plot of HCC-specific miRNAs. **(C)** Volcano plot of HCC-specific lncRNAs. **(D)** The hierarchical clustering heatmaps of HCC-specific mRNAs. **(E)** The hierarchical clustering heatmaps of HCC-specific miRNAs. **(F)** The hierarchical clustering heatmaps of HCC-specific lncRNAs.

A total of 420 samples including 370 HCC tumor tissues and 50 adjacent non-HCC tissues were included in our study from TCGA. To select the DEmRNAs, DEmiRNAs, and DElncRNAs in HCC, the edge R package was applied to compare the difference of expression levels of mRNAs, miRNAs, and lncRNAs in HCC with those in adjacent HCC tissues. On the basis of the cut-off criteria, mRNAs of 1,650 differentially expressed (fold change >2 and *p* < 0.05) were included finally. 1,467 were upregulated and 183 were downregulated; of 52 differentially expressed miRNAs (fold change >1 and *p* < 0.05), 44 were upregulated and eight were downregulated; of the 947 differentially lncRNAs that were identified between HCC tissues and normal tissues (fold change >2 and *p* < 0.05), 887 were upregulated and 60 were downregulated. The top 10 DEmRNAs, DEmiRNAs, and DElncRNAs are shown in Table 1 **.** In order to display the distribution of all differentially expressed RNAs intuitively in the dimensions of log(fold-change) and average log CPM, volcano plots were displayed (Figures 1A–C). The heatmap of clustering analysis was also used to show all RNAs which were differentially expressed between HCC tissues and adjacent non-HCC tissues (Figures 1D–F).

**TABLE 1:** Lists of top 10 DEmRNAs, top 10 DEmiRNAs and top 10 DElncRNAs.

Gene symbol	logFC	logCPM	Pvalue	FDR	Stage
Top 10 DEmRNAs					
CLEC4M	−5.48137	2.168058	7.47E-51	3.01E-48	Dwon
CLEC4G	−5.2942	3.455576	3.37E-80	5.56E-77	Dwon
CLEC1B	−5.10082	1.646952	7.38E-56	3.69E-53	Dwon
BMP10	−4.79512	-1.92181	2.52E-33	2.64E-31	Dwon
FCN2	−4.7926	3.300253	2.58E-70	2.66E-67	Dwon
REG1A	10.48096	6.990592	2.56E-16	3.15E-15	UP
LGALS14	10.49044	1.922612	1.06E-09	5.04E-09	UP
PGC	10.59397	6.836034	2.83E-19	5.06E-18	UP
REG1B	10.6009	3.154263	4.47E-11	2.63E-10	UP
REG3G	11.34777	2.78902	1.1E-09	5.23E-09	UP
Top 10 DEmiRNAs					
has-mir-4686	−2.59166	11.36234	4.03E-19	6.83E-16	Dwon
has-mir-6087	−1.75133	11.27664	7.99E-06	0.000558	Dwon
has-mir-514b	−1.62823	11.51238	1.69E-06	0.000143	Dwon
has-mir-8071-1	−1.40716	12.17483	0.000175	0.007596	Dwon
has-mir-7160	−1.25744	11.24068	2E-05	0.001207	Dwon
has-mir-548aa1	2.253896	12.02784	1.81E-09	4.31E-07	UP
has-mir-5707	2.351093	11.52312	0.000672	0.022297	UP
has-mir-3131	2.672176	12.20498	2.04E-09	4.31E-07	UP
has-mir-3125	3.351101	12.32628	6.97E-08	9.83E-06	UP
has-mir-483	3.66922	12.48561	6.11E-06	0.00045	UP
Top 10 DElncRNAs					
CH507-513H4.4	−4.41614	3.764086	5.91E-15	1.75E-13	Dwon
RP11-685F15.1	−4.318	4.289713	5.85E-26	1.35E-23	Dwon
AC006960.7	−3.95626	3.594539	5.62E-22	6.2E-20	Dwon
LINC01093	−3.63058	10.10333	3.12E-43	7.46E-40	Dwon
RP11-2N1.2	−3.53404	3.386575	6.89E-29	2.91E-26	Dwon
AC005150.1	7.115589	6.743932	4.22E-10	4.45E-09	UP
MAFA-AS1	7.140879	6.165614	1.68E-18	1.02E-16	UP
RP11-495P10.8	7.501832	8.341691	1.21E-21	1.19E-19	UP
RP11-402P6.9	7.630082	6.172778	4.83E-13	1.02E-11	UP
LINC01419	8.053134	10.51447	2.1E-15	7.03E-14	UP

### Functional enrichment analysis

**FIGURE 2:**
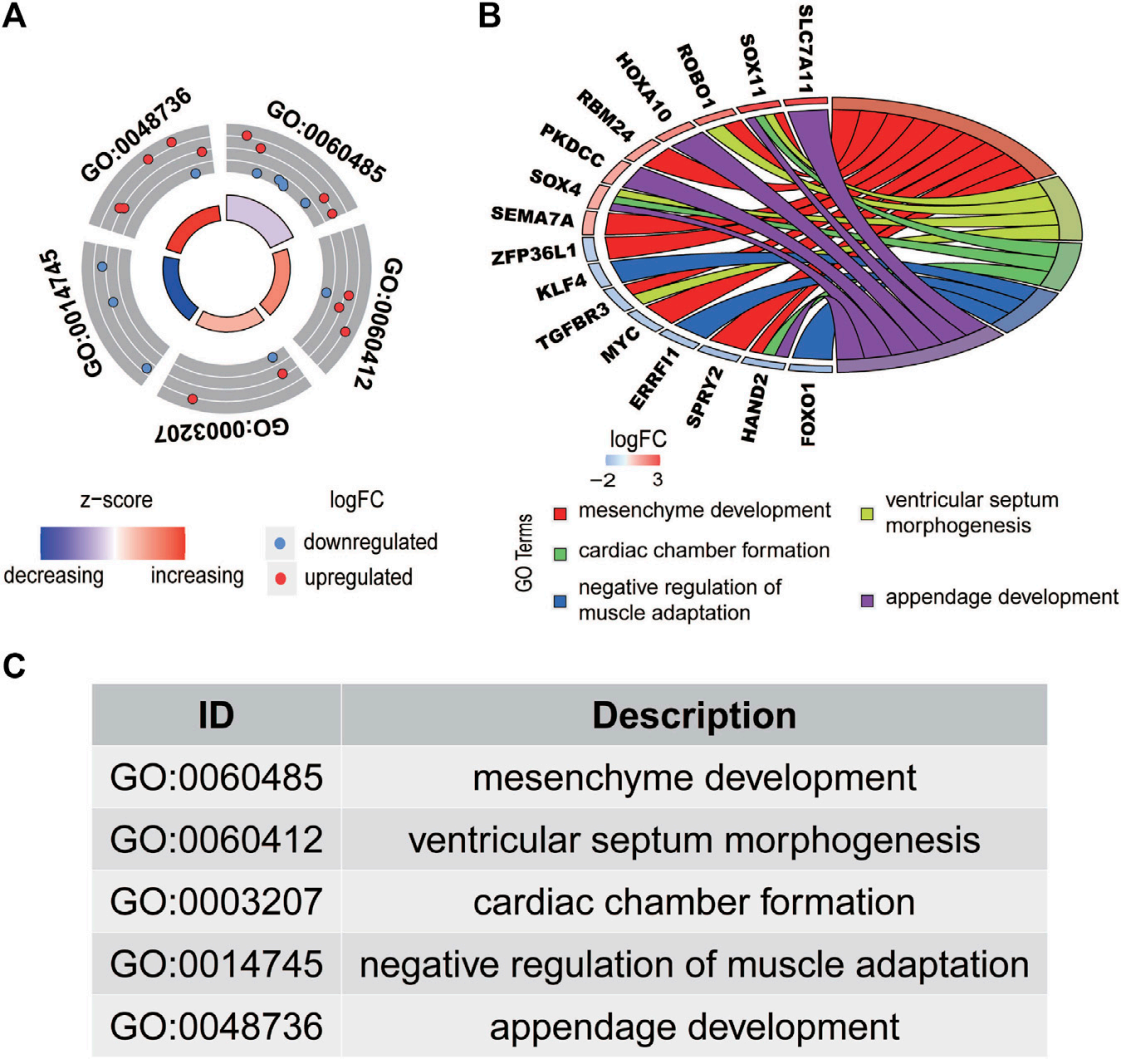
Functional enrichment analysis of differentially expressed mRNAs in HCC. The outer circle represents the expression (logFC) of differentially expressed mRNAs in each enriched gene ontology (GO) term. The red dots on the each GO term indicate the upregulated differentially expressed mRNAs, and the blue dots indicate the downregulated differentially expressed mRNAs. **(A)** The inner circle indicates the significance of the GO terms (log10-adjusted p values). **(B)** The circle indicates the correlation between the statistically top 16 differentially expressed mRNAs and their gene ontology terms. The top five GO terms are shown in **(C)**.

A total of 1,650 differentially expressed genes were analyzed with the GO database, which showed that 151 GOs based on statistical differences (*p* < 0.05). The highest biological process of GO is: “GO:0060485 mesenchyme development” (Figure 2A).

**FIGURE 3:**
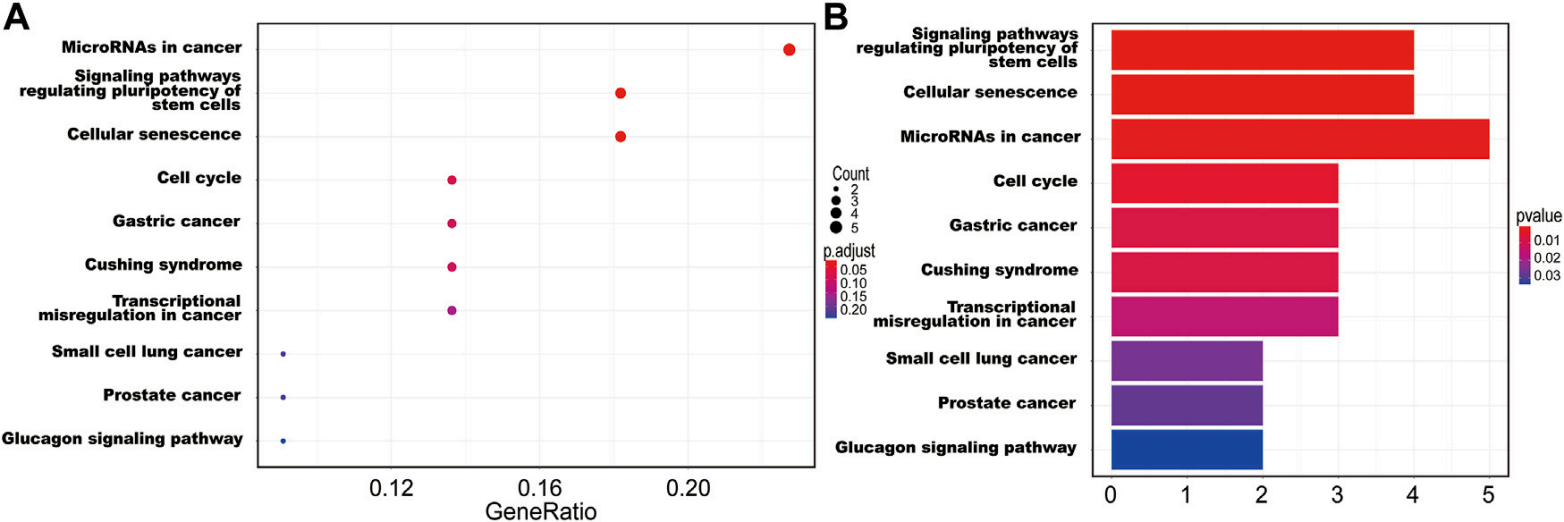
The ten main KEGG pathway enrichment of mRNAs included in the ceRNA network. **(A)** Bubble chart; **(B)** bar graph.

**FIGURE 4:**
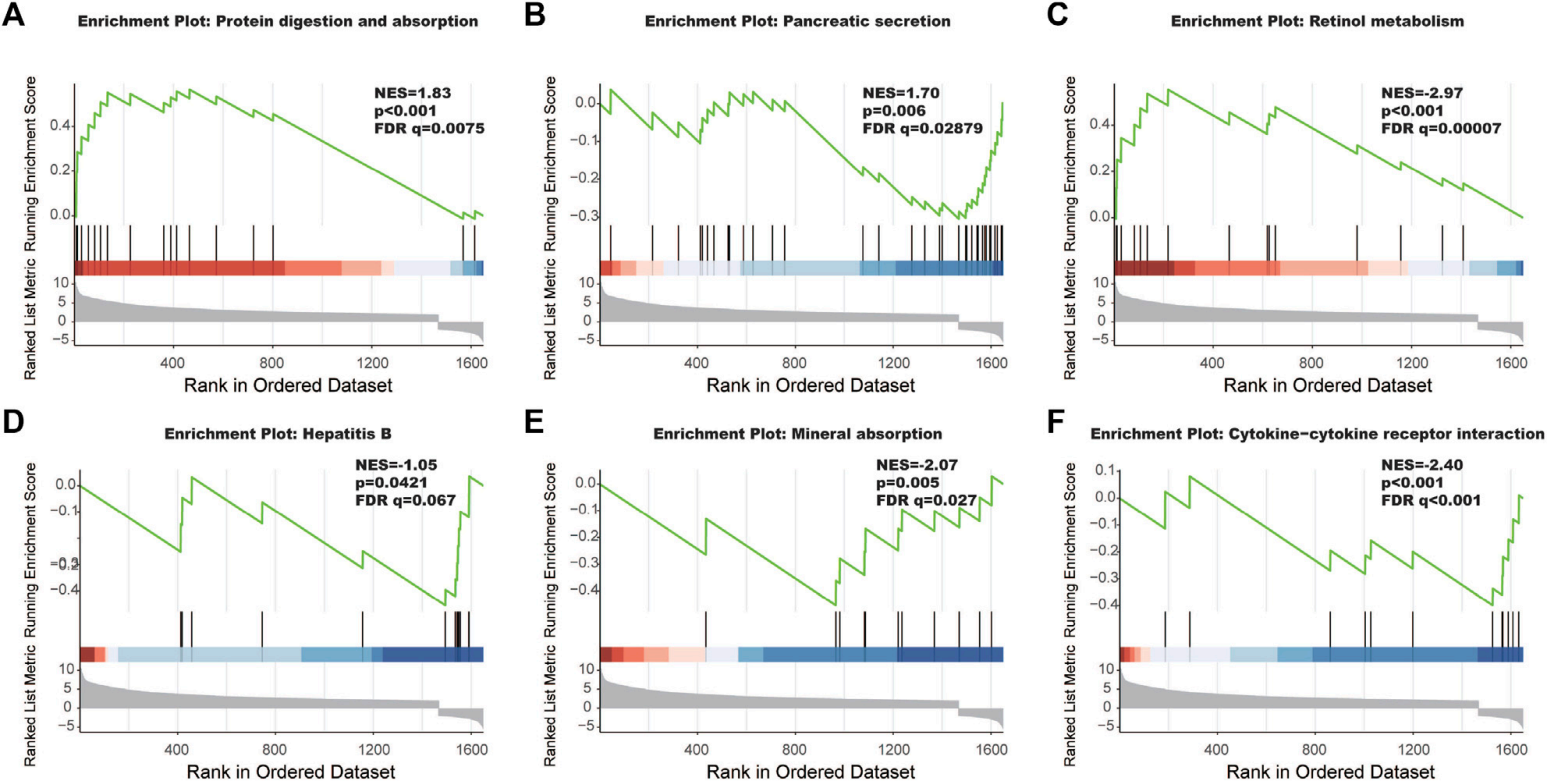
The most significantly enriched KEGG pathways between tumor tissues and adjacent non-tumor tissues. **(A)** Protein digestion and absorption, **(B)** pancreatic secretion, **(C)** retinol metabolism, **(D)** hepatitis B, **(E)** mineral absorption, and **(F)** cytokine-cytokine receptor interaction.

The interaction between the statistically significance top 16 mRNAs and their associated GO terms is shown in Figure 2B. The top five GO terms are shown in Figure 2C. In the result of analysis of the KEGG database, 64 pathways of DEmRNAs were appraisal, the most importantly ten pathways of which are displayed in Table 2; Figures 3A,B. The functional enrichment analysis of the ceRNA network was performed by gene set enrichment analysis (GSEA) to compare in HCC. The KEGG pathway enrichment analysis revealed that Protein digestion and absorption, Pancreatic secretion, Retinol metabolism, Hepatitis B, Mineral absorption, and Cytokine−cytokine receptor interaction were significantly related to the tumorigenesis and development of HCC (Figures 4A–F).

**TABLE 2:** mRNA pathway analysis in hepatocellular carcinoma.

Path ID	Pathway name	Diff/count	FDR
hsa04550	Signaling pathways regulating pluripotency of stem cells	4	0.026804541
hsa04218	Cellular senescence	4	0.026804541
hsa05206	MicroRNAs in cancer	5	0.029550252
hsa04110	Cell cycle	3	0.082848778
hsa05226	Gastric cancer	3	0.098166222
hsa04934	Cushing syndrome	3	0.098166222
hsa05202	Transcriptional misregulation in cancer	3	0.150461687
hsa05222	Small cell lung cancer	2	0.226946745
hsa05215	Prostate cancer	2	0.226946745
hsa04922	Glucagon signaling pathway	2	0.244767502

### Interactions between mRNAs, miRNAs, and lncRNAs

**FIGURE 5:**
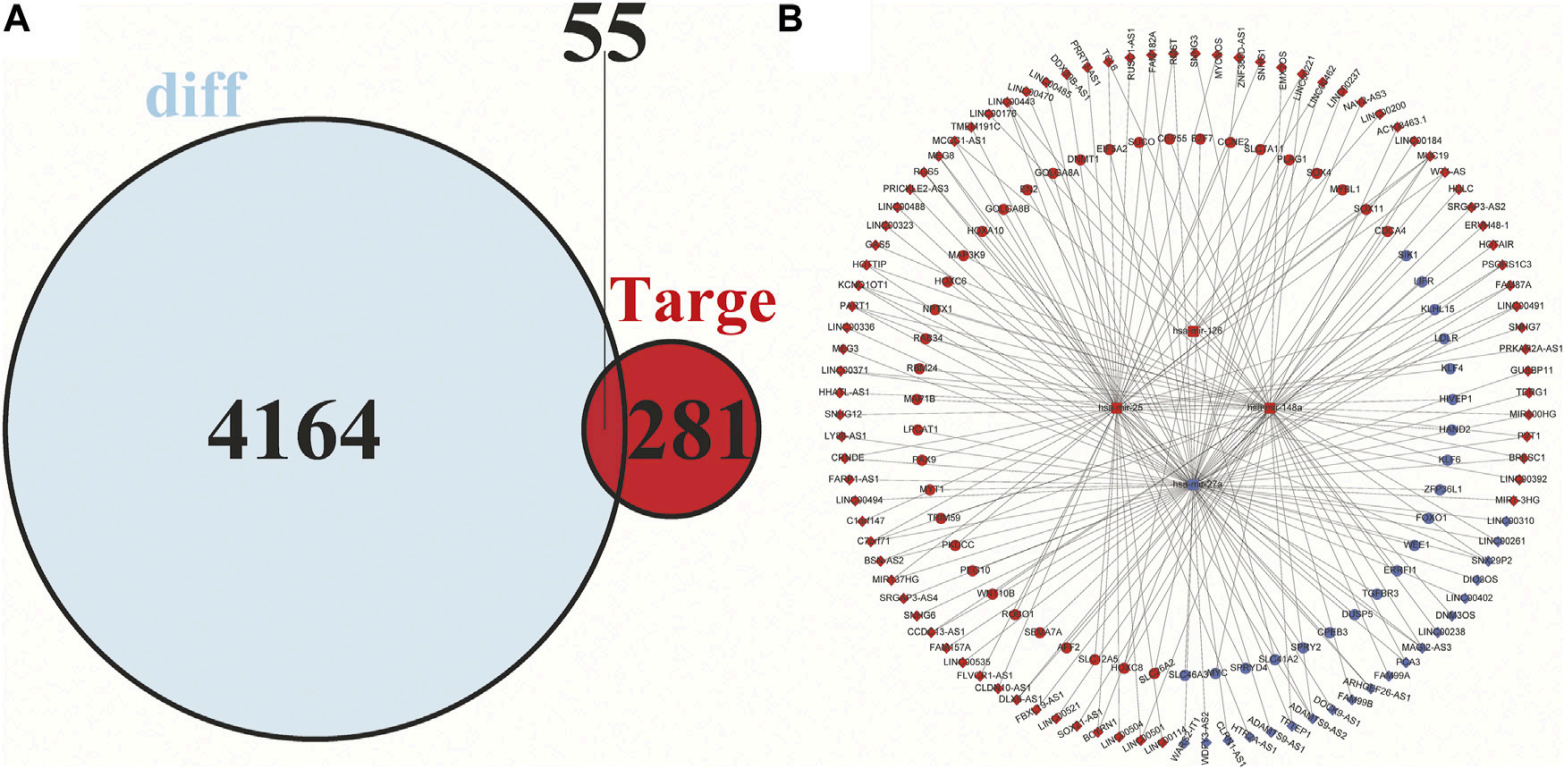
Venn diagram of mRNAs included in the ceRNA network and construction of a lncRNA-miRNA-mRNA network in HCC. **(A)** The red area represents the number of differentially expressed mRNAs, and the blue area shows the number of targets of differentially expressed mRNAs. The overlapping area in the middle indicates the number of mRNAs that both belonged to the differentially expressed mRNAs and the target mRNAs. **(B)** The lncRNAs, miRNAs, and mRNAs are represented by diamonds, rounded rectangles, and circles, respectively. The red nodes indicate high expression, and the blue nodes indicate low expression.

Based on the miRDB, miRTarBase and TargetScan databases, a total of 349 pairs of miRNA-mRNA regulate relationship were predicted. Similarly, 146 pairs of interactions between lncRNAs and miRNA were predicted by the miRcode database. After the two are combined, 15,795 pairs of lncRNA-miRNA-mRNA interactions were acquired. Finally, 99 DElncRNAs, four DEmiRNAs and 55 DEmRNAs were included in the constructed ceRNA network Figure 5A.

### Competing endogenous RNA network analysis

Cytoscape v3.8.2 was applied to visualize the competing endogenous RNA (ceRNA) network. In the ceRNA network, 99 DElncRNAs, four DEmiRNAs, and 55 DEmRNAs were included, which is shown in Figure 5B.

### Identification of survival-related RNAs in the ceRNA network in HCC

**FIGURE 6:**
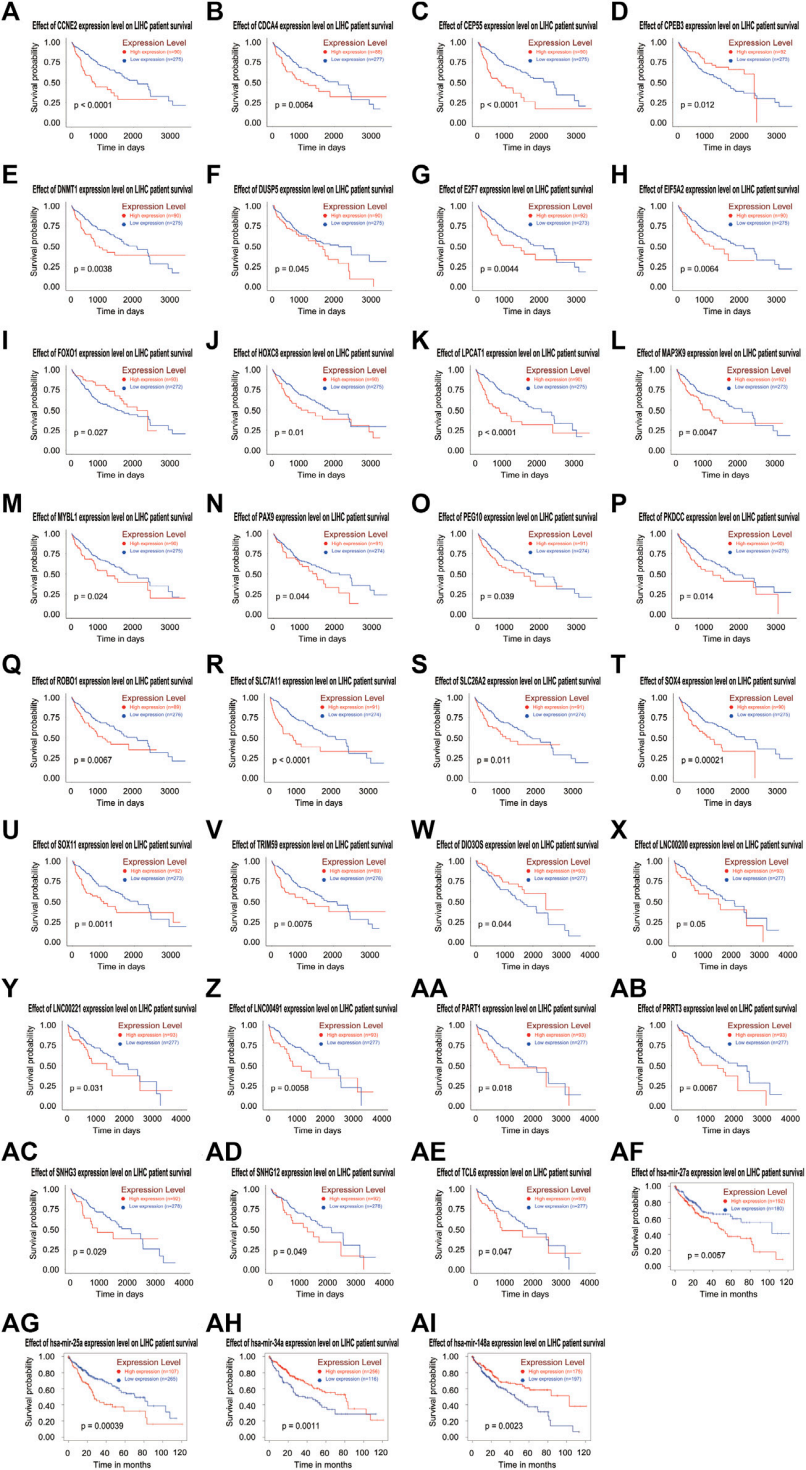
Based on the ceRNA network, Kaplan-Meier curve analysis of DElncRNAs, DEmRNAs, DEmiRNAs, and overall survival rate in HCC samples (A-AI).

**FIGURE 7:**
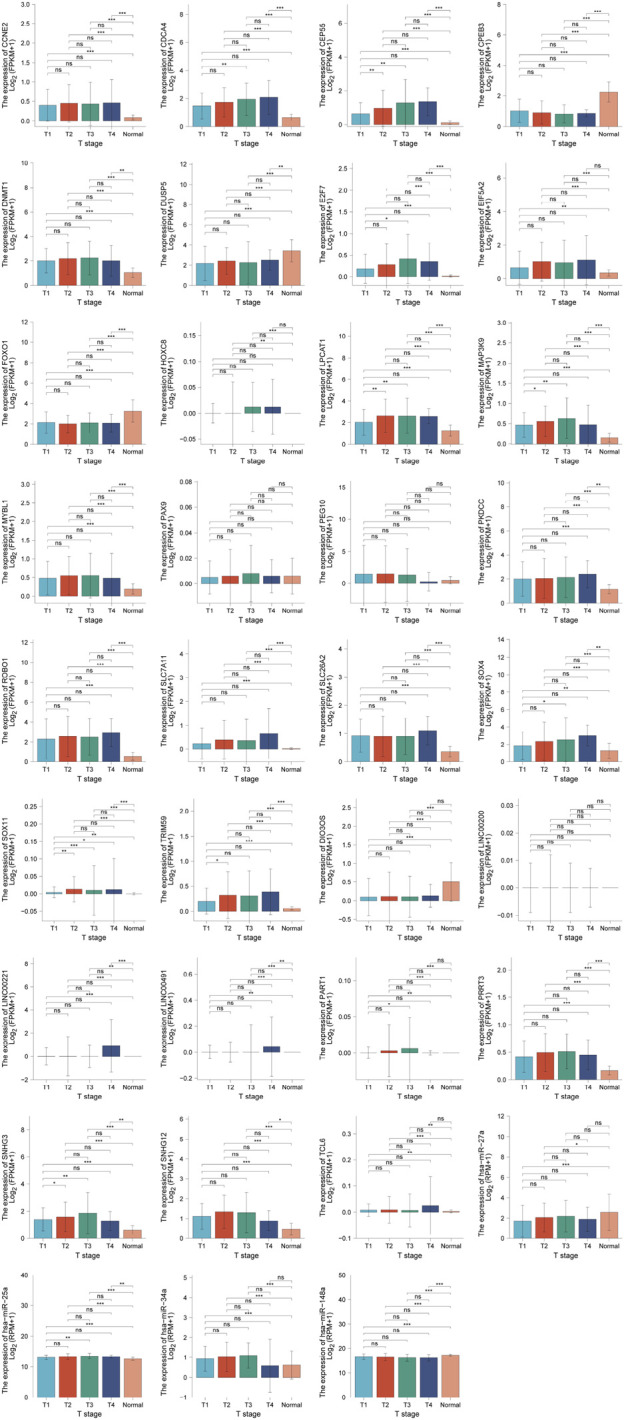
Correlation between ceRNA and tumor stage.

In order to clarify the prognostic characteristics of mRNAs, lncRNAs, and miRNAs in the ceRNA network, based on the TCGA data on the UALCAN website (http://ualcan.path.uab.edu/cgi-bin/ualcan-res.pl) and Kaplan-Meier Plotter (https://kmplot.com/analysis/index.php?p=service), we evaluated the association between the expression of lncRNA, miRNA, mRNA, and overall survival in HCC patients. Survival analysis confirmed 22 DEmRNAs, four DEmiRNAs (has-mir-27a, has-mir-34a, has-mir-25 and has-mir-148a) and nine DElncRNAs that were associated with poor patient prognosis in HCC. The patient survival curves of the relationships between lncRNAs, mRNAs, and miRNAs are shown in Figure 6. Based on these differentially expressed genes, futher exploration was performed to assess the correlation of them with tumor stage, which are showed in Figure 7.

### Construction of a predictive model of seven lncRNAs in HCC patients

The univariate Cox regression analysis was used to determine the risk factors related to patient prognosis, and 89 lncRNAs that were conspicuously associated with OS (*p* < 0.05) were incorporated. Next, the result of the lasso cox regression was applied to multivariate cox regression to identify the final independent prognosis risk factors in HCC. Finally, a model was constructed to predict the prognosis of HCC patients. The predictive model is described as survival risk score = (0.00937  ×  expression value of AATK_AS1 + 0.02378  ×  expression value of AC114803.3 + 0.00826 × expression value of AC008271.1 + 0.05058 × expression value of RP1_287H17.1 + 0.01554 × expression value of LINC01060 + 0.019146 × expression value of RP11.501O2.5 + 0.000542 × expression value of LINC00942 + 0.050611 × expression value of RP11.282I1.1 + 0.004048 × expression value of RP11.314M24.1 + 0.025259 × expression value of LINC01060 + 0.01642 × expression value of CTD_2532K18.2 + 0.00059 × expression value of MAFG_AS1). The multivariate Cox analysis is shown in Table 3.

**TABLE 3:** Multivariate cox regression analysis of 16 prognostic lncRNAs associated with overall survival in HCC patients.

LncRNA	Coef	HR	95%CI		*p*-value
			Low	Up	
AATK_AS1	0.00059	1.00942	1.00309	1.01579	0.00350
AC114803.3	0.02378	1.02406	0.99912	1.04962	0.05875
AC008271.1	0.00826	1.00830	1.00232	1.01431	0.00646
RP1_287H17.1	0.05058	1.05188	1.02619	1.07821	0.00006
LINC01060	0.01554	1.01566	1.00981	1.02155	<0.00001
CTD_2532K18.2	0.01642	1.01656	0.99954	1.03387	0.05658
MAFG_AS1	0.00059	1.00059	1.00005	1.00112	0.03102

### Risk groupings and ROC analysis

**FIGURE 8:**
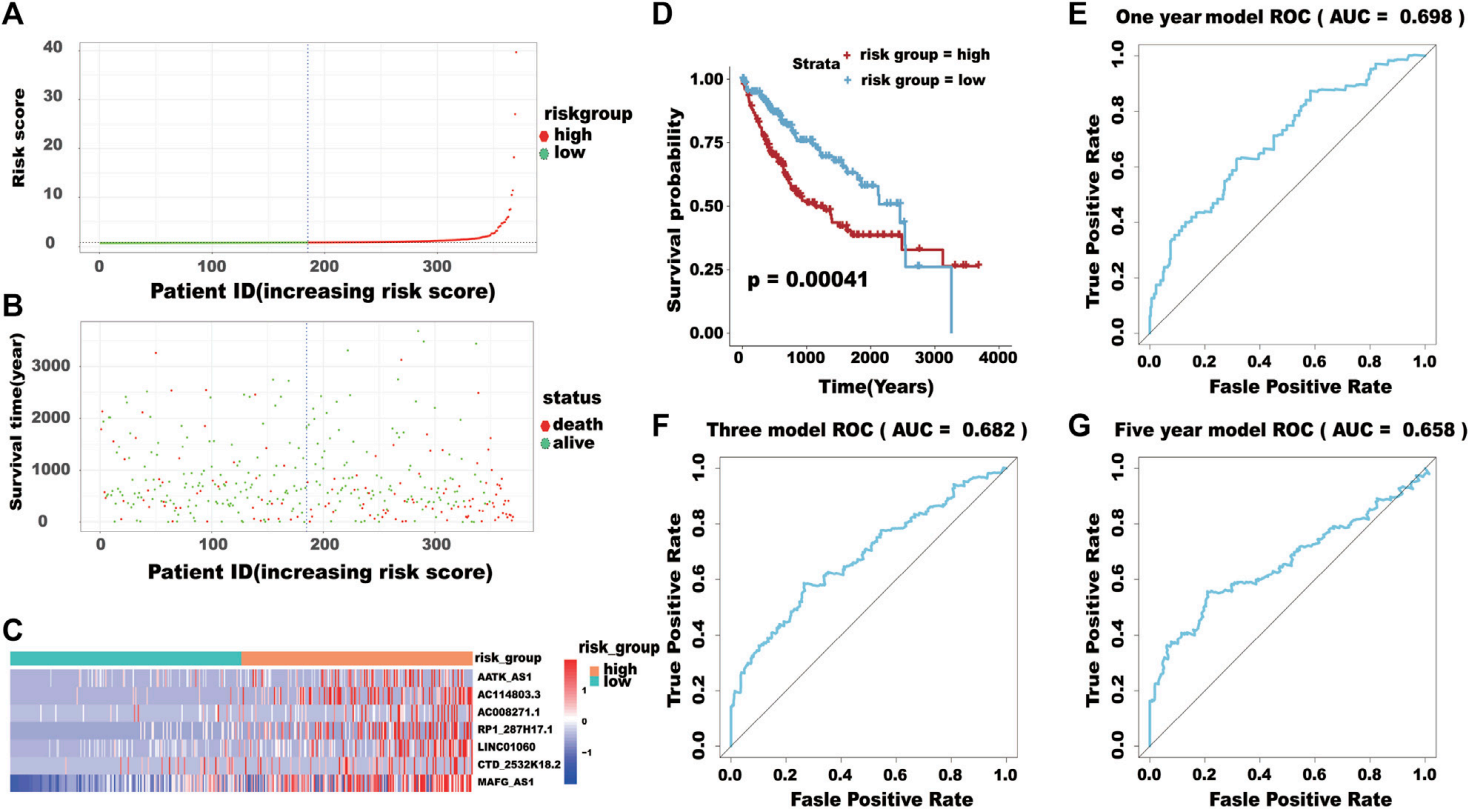
**(A)** The distribution of the high-risk group and low-risk group. **(B)** Survival status of the high-risk and low-risk groups. **(C)** The heatmap of the expression levels of the seven lncRNAs. **(D)** Kaplan-Meier curve analysis for the overall survival (OS) of HCC patients using the seven lncRNA signatures. **(E–G)** ROC curve analysis of the prognostic (1-, 3-, 5-year) seven lncRNA signatures.

Based on the model of survival risk score, the 370 HCC patients with complete survival information were divided into a high-risk group (*n* = 185) and the other for low-risk group (*n* = 185) by median risk scores (Figure 8A). It is clear that the mortality of higher risk scores patients was significantly higher than that of lower risk scores patients (Figure 8B). There was significant difference in the expression levels of the eight genes between the two groups (Figure 8C). The Kaplan–Meier curve with a log-rank statistical examination showed shorter high-risk group patient survival, which is shown in Figure 8D. The ROC curve was analyzed to test the influence on the 16-lncRNA signature associated with OS (1-, 3-, and 5-year) in HCC (Figures 8E–G).

## Discussion

HCC is the leading cause of cancer-related death worldwide (Yu, 2016). LncRNAs, function as ceRNAs by binding miRNAs, have been reported to be involved in the physiological and pathological processes of various diseases (Zou et al., 2020). At present, a large number of studies indicate that the ceRNA network is of great significance in the development and progression of tumors (Qi et al., 2015). For instance, some methodical analysis of the ceRNA network has been performed in many cancers, such as pancreatic cancer, breast cancer, and lung cancer (Tay et al., 2014; Li et al., 2016; Zhou et al., 2016). However, the comprehensive analysis of lncRNA-mediated ceRNA regulatory network in HCC remains scarce.

In this study, we tried to find out the potential implications of lncRNAs in the diagnosis and prognosis of HCC, explore the possible regulatory mechanisms and functional roles of lncRNAs as ceRNAs in the progression of HCC are going to be crucial. In this study, we used the data of HCC from TCGA database to generate the competitive endogenous RNAs network, which the lncRNA and mRNA sharing a common miRNA according to the ceRNA theory. This novel lncRNA–miRNA–mRNA ceRNA network of HCC, which was comprised of nine lncRNA nodes, four miRNA nodes, 22 mRNA nodes, and 15,795 edges. GO analysis displayed that genes in our ceRNA regulatory network were involved in the regulation of mesenchyme development, ventricular septum morphogenesis, cardiac chamber formation, negative regulation of muscle adaptation, and appendage development. Some studies declared that non-coding RNAs could regulate the progression cancer cells via regulating the Wnt/beta-catenin signal. Not only that, the Wnt/beta-catenin pathway could also promote cancer stem cell self-renewal, metastasis, and chemoresistance in all types of epithelial ovarian cancer (Nguyen et al., 2019). Many studies have focused on the signaling pathway in tumor (Gao et al., 2017; Ram Makena et al., 2019), PI3K-Akt signaling pathway and Focal adhesion pathway (Kanteti et al., 2016; Ebrahimi et al., 2017), indicating that those pathways are closely related to the tumorigenesis and metastasis of HCC. Therefore, the tumor-related pathway is an important biological process, which not only carries a variety of biological functions but also is closely related to the development and occurrence of many disease processes, especially in tumor.

Among the 99 lncRNAs in the ceRNAs, six lncRNAs were found to have a significant impact on the prognosis of HCC. DIO3OS is an antisense lncRNA transcribed from the DIO3 gene-imprinted locus (Hernandez et al., 2004). Previous studies have shown that DIO3OS is associated with multiple tumors, such as thyroid cancer, pancreatic cancer and liver cancer (Cui et al., 2019; Wang et al., 2020; Wang et al., 2021). LINC00200 is a functionally uncharacterized novel lncRNA. There are studies suggested that LINC00200 could be used as a candidate molecular marker for the diagnosis of gastric cancer, and new insights were subsequently provided for gastric cancer treatment (He et al., 2021). Studies have shown that LINC00221 may promote the progression of HCC through the mediation of the let-7a-5p/MMP11 axis (Yang et al., 2021). LINC00491 is a lncRNA that has been validated as an oncogene which promotes tumor progression of colon adenocarcinoma (Wan et al., 2019). PART1 has been reported to be an oncogenic factor in HCC (Lv et al., 2018; Ye et al., 2019). PRRT3-AS1 silencing can upregulate apoptosis and autophagy while diminishing the proliferation, migration, and invasion of prostate cancer cells via the mTOR signaling pathway (Fan et al., 2020). Recent studies have showed that SNHG12 can regulates the Wnt/β-catenin signaling pathway in papillary thyroid carcinoma cells to promote cellular proliferation and metastasis, but also plays a regulate the occurrence and development of glioma (Liu et al., 2018), osteosarcoma (Zhou et al., 2018) and gastric carcinoma (Yang et al., 2018). Nevertheless, the prognostic role and underlying molecular mechanisms of TCL6 and SNHG3 in HCC is unclear. Therefore, the ceRNA network was reliable.

Based on DElncRNAs, a model was constracted to predict the prognosis of HCC patients. This model consists of 16 genes, including AC016735.2, RP11.1C1.6, RP11.730G20.1, AATK.AS1, LINC00632, RP11.501O2.5, LINC00942, RP11.282I1.1, RP11.314M24.1, LINC01060, DUXAP8, RP1.287H17.1, LINC01532, AC008271.1, CTC.327F10.5, and RP11.109J4.1. Among them, LINC01532 was associated with low risk of poor prognosis, and the remaining genes were associated with high risk of poor prognosis.

The significance of this study lies in not only constructing a novel ceRNA hub regulatory network through utilizing TCGA database but also screening out lncRNAs associated with HCC prognosis via integrating clinical data. What is more important is that we constracted a prognosis model based on TCGA databases. However, this research also has some limitations because it mainly relies on analysis of sequencing data and public clinical data and requires further experimental explorations. The relationship of other clinical features (including TNM stage, the size and number of tumor) to ceRNA should be investigated. At the same time, this model is also need to be verified by inside and outside.

## Conclusion

In the current study, we constructed a ceRNA network to predict the progression of HCC using the RNA transcriptome sequencing data from TCGA database. The application of public genomic data might assist in identifying potential therapeutic targets and provide novel methods for investigating the functions and mechanisms of ceRNAs in malignant tumors.

## Data Availability

The datasets presented in this study can be found in online repositories. The names of the repository/repositories and accession number(s) can be found in the article/Supplementary Material.
